# Long-term dynamics of tropical heath forests in Brunei Darussalam: forest structure, tree demography and community reassembly over 30 years

**DOI:** 10.3897/BDJ.14.e194507

**Published:** 2026-06-30

**Authors:** Irsalina Syakirah Mohd Ikbal, Salwana Md Jaafar, Norhayati Ahmad, Rahayu Sukmaria Sukri

**Affiliations:** 1 Asian School of the Environment, Nanyang Technological University, Singapore, Singapore Asian School of the Environment, Nanyang Technological University Singapore Singapore; 2 Institute for Biodiversity and Environmental Research, Universiti Brunei Darussalam, Bandar Seri Begawan, Brunei Institute for Biodiversity and Environmental Research, Universiti Brunei Darussalam Bandar Seri Begawan Brunei

**Keywords:** Forest disturbances, forest dynamics, forest management, forest succession, *Kerangas* forest

## Abstract

This study presents the first long-term analysis forest dynamics in lowland Bornean heath forests in Brunei Darussalam, based on two 0.96 ha permanent forest plots, established in 1992 at Bukit Sawat Forest Reserve and Badas Forest Reserve. The plots were re-censused in 2022, during which all trees with stem diameter ≥ 5 cm were tagged, identified and measured for their stem diameter and basal area. Over the 30-year period, mean stem abundance and mean tree density significantly decreased, while mean stem diameter and mean basal area significantly increased, at both heath forest sites. At Bukit Sawat, mean species richness and diversity indices were significantly higher in 1992 than in 2022, but no significant temporal dfferences were observed at Badas. No significant differences between sites were detected in mean annual diameter growth rate, mortality and recruitment rates. Notably, both plots exhibited significant shifts in tree community composition over the 30-year period, coupled with a 24% increase in total species richness at Bukit Sawat and a 4% decrease at Badas. Despite their locations in protected areas, droughts events and anthropogenic disturbance over the past three decades likely contributed to observed changes in forest structure, species diversity and community composition. These findings provide important insights into the long-term dynamics of threatened Bornean heath forests and underscore the need for continued monitoring to guide conservation and management strategies.

## Introduction

Long-term studies on tropical tree communities are essential in understanding forest dynamics and community responses. The use of permanent long-term research plots in tropical forests (Davies et al. 2021, ForestPlots.net 2021) has significantly contributed to the body of knowledge on species co-existence and diversity and ecosystem functioning of tropical forests. Ecological research and monitoring programmes, such as monitoring of species diversity and tree compositions in protected forest areas (Lindenmayer et al. 2012, Gardner et al. 2012, Watson et al. 2018), have also facilitated effective management and forest conservation (Carey et al. 1994, Sulistyawati et al. 2022). Forest plots provide ideal settings for long-term studies of the drivers of tropical forest species distributions and co-existence, enabling improved understanding of how forest communities are structured at both spatial and temporal scales (Davies et al. 2021) and how climate change can impact tropical forests (Qie et al. 2017).

Tree community composition of hyperdiverse Bornean tropical lowland forests are well-documented and several long-term studies on tree communities within permanent plots have been conducted. Despite extensive studies on Bornean tree communities, lowland Bornean (*Kerangas*) heath forests are understudied (Ikbal et al. 2023, Anirudh et al. 2025). These unique forests occur on acidic, nutrient-poor sandy soils (Proctor et al. 1983, Jaafar et al. 2016) and are characterised by their stunted vegetation, densely-packed pole-sized trees and sclerophyllous leaves (Ghazoul and Sheil 2010, Ashton and Lee 2022). In Brunei Darussalam, heath forests cover approximately 3455 ha (~ 0.6% of forest area), occurring as patches along the coastline or inland adjacent to mixed dipterocarp or peat swamp forests (Wong and Kamariah 1999, Ikbal et al. 2023, Ikbal et al. 2023). Several studies have described the floristic composition and forest structure of Brunei's heath forests (Davies and Becker 1996, Din et al. 2015, Ikbal et al. 2024), but no long-term study on its forest dynamics and changes in tree communities has been attempted. Brunei's intact heath forests are of conservation significance given their high levels of plant endemism (Ashton 2009) and long-term studies of their dynamics are crucial to better inform management action. 

This study investigated growth, recruitment, mortality and changes in tree community composition in two 0.96 ha heath forest plots in Bukit Sawat and Badas, Brunei Darussalam (Davies and Becker 1996) over a 30-year period (1992 - 2022). Two hypotheses were developed: (1) For each of the 0.96 ha plot, significant changes in tree communities and forest structure will be recorded over the last 30 years and (2) mortality, recruitment and growth rates of tree species over the last 30 years will differ between the heath forests at Bukit Sawat and Badas.

## Material and methods

### Site description

**Figure 1.**
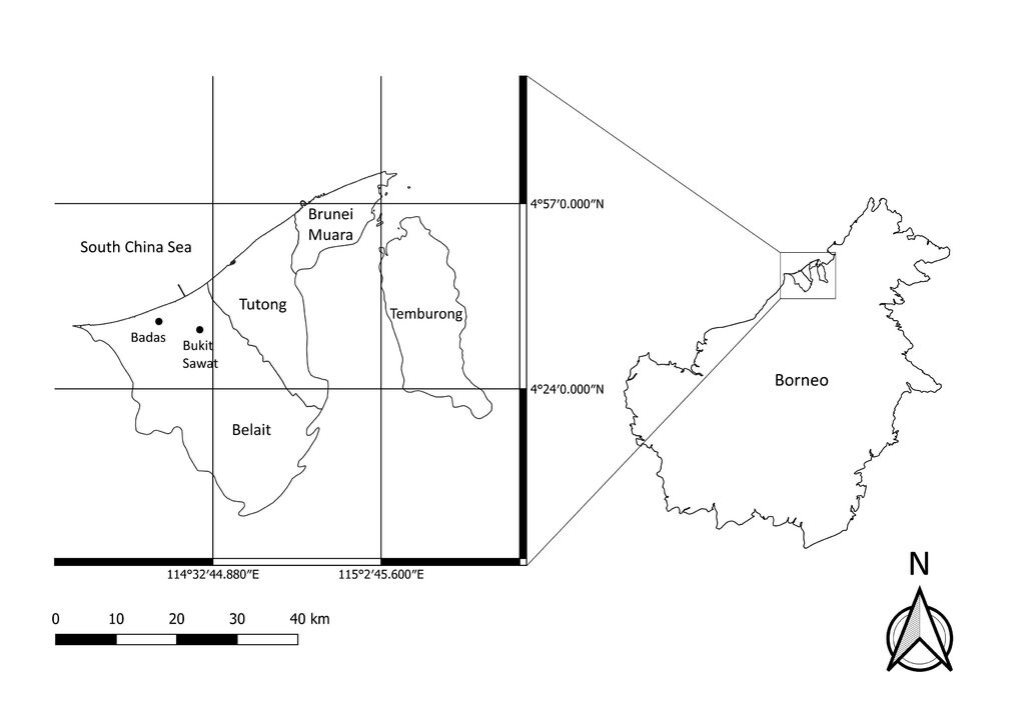
Location of study sites at two 0.96 ha permanent heath forest plots in Bukit Sawat and Badas, Belait District, Brunei Darussalam.

The study was carried out in two permanent heath forest plots (0.96 ha each; Fig. 1), located in the Belait District, Brunei Darussalam and maintained by the Institute for Biodiversity and Environmental Research, Universiti Brunei Darussalam. The sites comprise Bukit Sawat Forest Reserve (4°34'32.27"N, 114°30'24.73"E, 11-23 m a.s.l.) and Badas Forest Reserve (4°56'72.51"N, 114°41'76.45"E, 11-16 m a.s.l.). Both plots were originally established in 1992 as part of a long-term forest dynamics monitoring programme (see Davies and Becker (1996)). Despite their similar plot size and shared monitoring history, the two sites differ markedly in environmental conditions and vegetation structure. Bukit Sawat is characterised by gently uneven terrain interspersed with poorly drained, waterlogged patches, supporting a mixed assemblage of heath and peat swamp forest species. In contrast, the Badas site is situated on a peat dome (Nafiah et al. 2022) with minimal variation in elevation and is dominated by typical heath forest taxa, particularly *Agathis borneensis* (Araucariaceae).

The climate of Brunei Darussalam is equatorial, characterised by high temperatures, humidity and rainfall throughout the year. Mean annual temperatures typically range between 26 to 28°C, while annual rainfall generally exceeds 2500 mm. Rainfall is influenced by the northeast and southwest monsoon systems, with wetter conditions typically occurring between October and January. During the study period (1992 to 2022), several El Niño events, including the severe 1997–1998 and 2015–2016 events, resulted in periods of reduced rainfall and drought across Borneo (Walsh and Newbery 1999, Potts 2003).

### Tree census and species identification

The first tree census within these plots was carried out in 1992 by Davies and Becker (1996) in which all living trees with diameter at breast height (DBH) ≥ 5 cm were tagged and measured. Voucher specimens from the 1992 census were collected, identified and deposited in the Universiti Brunei Darussalam Herbarium (Davies and Becker 1996). To assess growth, recruitment, mortality and changes in tree diversity and community composition over a 30-year period, both 0.96 ha plots were re-censused in 2022 (Ikbal et al. 2024). Each 0.96 ha plot was divided into 20 x 20 m sub-plots following the original grid system of Davies and Becker (1996). All living trees in each subplot at both sites with diameter at breast height (DBH) ≥ 5 cm were tagged and measured using a diameter tape (Metri, Germany). DBH measurements followed the standard protocols of the Center for Tropical Forest Science (Condit 1998). Trees censused in 2022 were identified to species level in the field with assistance from the Brunei National Herbarium (BRUN) and voucher specimens were collected for confirmation. The full stem-level data for both plots are available upon request from the senior corresponding author (RSS).

### Statistical analyses

All data analyses were conducted using R version 3.6.3 (R Core Team 2020). To evaluate tree species diversity, the vegan package version 2.5-7 (Oksanen et al. 2020) in R v. 3.6.3 (R Core Team 2020) was used to calculate species richness and diversity indices (Shannon’s index, Inverse Simpson’s Index and Evenness) at the subplot level for both sites, separately for the 1992 and 2022 censuses. Species richness and diversity indices (Shannon’s Index, Inverse Simpson’s Index and Evenness) were compared for each 0.96 ha plot between the two censuses. Significant differences in species richness, abundance, tree density and diversity indices for each 0.96 ha plot between the two census periods were analysed using linear mixed effects (LME) models with nlme version 3.1-137. Where necessary, these parameters were either arcsine or log10 transformed prior to analysis. The factor “Census Year” was modelled as the fixed effect, forest structure measurements, stem abundance, tree density, species richness and diversity indices were modelled as the response variables and subplot number was modelled as the random effect. All LME model selections were based on the protocols by Zuur et al. (2009) and Pinheiro et al. (2018).

To evaluate forest structure, stem diameter measurements were used to determine DBH size class distributions and basal area of trees in different size classes. For each study site and tree census, basal area was calculated as follows (Avery and Burkhart 2015):

*Basal Area (B.A.)* = \begin{varwidth}{50in}\begin{equation*}
            \pi \left(\frac{DBH}{2}\right)^2
        \end{equation*}\end{varwidth}

In addition, trends in DBH size class distribution by site over each census period were compared.

To determine floristic similarity between forest types, PERMANOVA (Anderson 2008) was conducted using the *adonis* function in the R vegan package version 3.6.3 for species abundance data (Nafiah et al. 2022). The function *pairwise.adonis* was used to conduct pairwise comparisons (Martinez Arbizu 2020).

Annual tree diameter growth rates (cm yr^-1^) were calculated for individual trees using the absolute diameter growth rate (AGR) as follows (Swaine et al. 1987, Sawada et al. 2015):

\begin{varwidth}{50in}\begin{equation*}
            AGR = \frac{Final\ DBH - Initial\ DBH}{Census\ interval}
        \end{equation*}\end{varwidth}

where the final DBH is the DBH measurement (in cm) during the 2022 census, the initial DBH is the DBH measurement (in cm) during the 1992 census and the census interval is 30 years.

Mortality (m) and recruitment (r) rates for each site were calculated following Condit et al. (1999) as follows:

\begin{varwidth}{50in}\begin{equation*}
            m = \frac{\ln n_0 - \ln S_t}{t}
        \end{equation*}\end{varwidth}

\begin{varwidth}{50in}\begin{equation*}
            r = \frac{\ln n_t - \ln S_t}{t}
        \end{equation*}\end{varwidth}

where n_0_ is the initial number of stems recorded in the 1992 census, n_t_ is the total number of stems recorded in the 2022 census, S_t_ is the number of surviving stems recorded in the 2022 census and t is the census interval of 30 years. Mortality and recruitment rates were expressed as % yr^-1^ by multiplying with 100%.

Sub-plot level values of mean growth rates, mean % mortality and mean % recruitment were calculated and used to determine differences between the two heath forest sites using linear mixed effects (LME) models in R version 3.6.3 models (R Core Team 2020) with nlme version 3.1-137. Where necessary, these parameters were either arcsine or log10 transformed prior to analysis. The factor “Site” was modelled as the fixed effect, mean growth rates, mean % mortality and mean % recruitment were modelled as the response variables and subplot number was modelled as the random effect.

## Results

### Differences in abundance, tree density, stem diameter, basal area and size class distributions over a 30-year period

At both sites, the total abundance of trees with DBH ≥ 5 cm decreased over the 30-year period between 1992 and 2022 (Bukit Sawat, n = 1613 vs. 1317 trees; Badas, n = 1341 vs. 1052 trees). Mean stem abundances and mean tree density were significantly higher in 1992 than in 2022 at both sites (Table 1, Suppl. material 1). Mean DBH and mean basal area were significantly higher in 2022 than 1992, for both Bukit Sawat and Badas (Table 1, Suppl. material 1).

**Figure 2.**
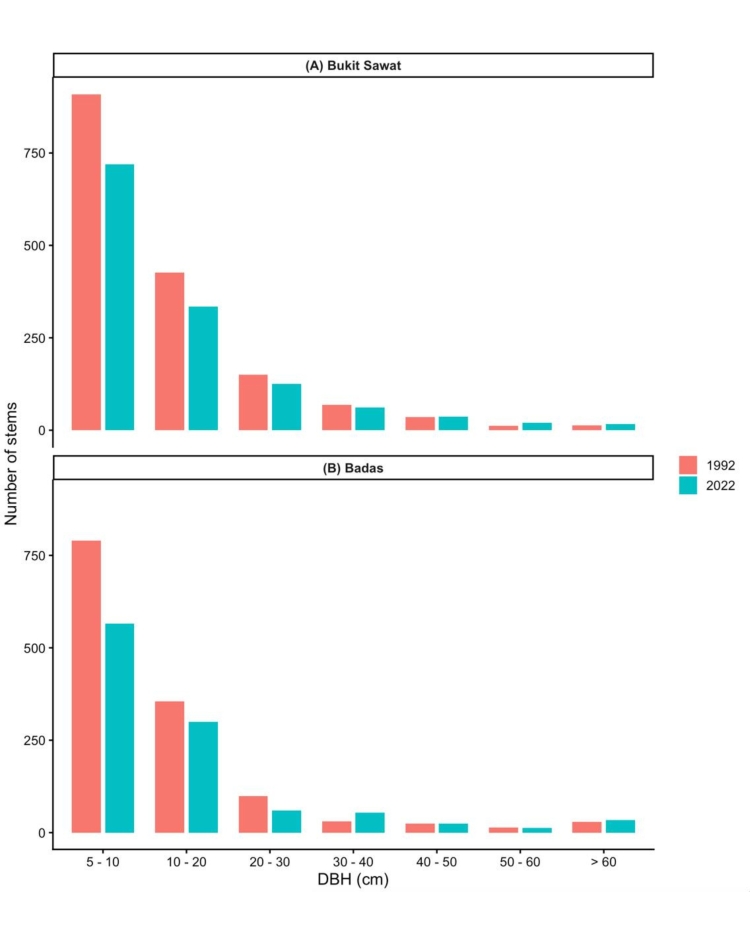
Size-class distributions of all trees with diameter at breast height, DBH ≥ 5 cm recorded during the 1992 and 2022 censuses, respectively, within two 0.96 ha permanent heath forest plots at Bukit Sawat (A) and Badas (B), Brunei Darussalam. Values represent the total number of stems recorded within each DBH class for each census period.

During both census periods, the size class distributions, based on DBH, followed reverse J-shaped distributions, with small-sized trees of DBH < 10 cm comprising > 50% of the stems recorded and representing the most abundant size class at both Bukit Sawat and Badas (Fig. 2). Over the 30-year period, there was a noticeable increase in the abundance of large trees with DBH ≥ 40 cm, particularly at Bukit Sawat (n = 59 vs. 72) and to a lesser extent at Badas (n = 67 vs. 72, Fig. 2).

### Differences in species richness, diversity and community compositions over a 30-year period

Bukit Sawat recorded higher total species richness in the 2022 census (n = 208 species) than in the 1992 census (n = 168 species; Table 2). In contrast, Badas recorded lower total species richness in the 2022 census (n = 108 species) compared to the 1992 census (n = 113 species; Table 2). The complete checklist of trees from the two census periods (Suppl. material 2) revealed that a total of 116 species were recorded in both census periods at Bukit Sawat, of which 53 were recorded exclusively in 1992 and 84 exclusively in 2022. For Badas, a total of 68 species were recorded in both census periods, of which 43 were recorded exclusively in 1992 and 39 exclusively in 2022.

At both sites, the most species-rich family remained unchanged over the 30-year period. Dipterocarpaceae was the most species-rich family in Bukit Sawat, increasing from 18 species in 1992 to 23 species in 2022, while Myrtaceae was the most species-rich family in Badas, increasing from 11 to 17 species. In Bukit Sawat, *Dipterocarpus borneensis* was consistently the most abundant tree species in both the 1992 and 2022 censuses, although its abundance declined from 99 to 80 individuals. In Badas, *Syzygium bankense* remained the most abundant species, declining from 285 individuals in 1992 to 194 individuals in 2022, followed by *Agathis borneensis*, which declined from 217 to 151 individuals.

For Bukit Sawat, mean species richness and diversity indices (Shannon’s Index, Inverse Simpson’s Index) were significantly higher in the 1992 census than the 2022 census, but mean Evenness did not significantly differ (Table 2). In contrast, no significant differences were detected in mean species richness and mean diversity indices between census years in Badas (Table 2, Suppl. material 3).

**Figure 3.**
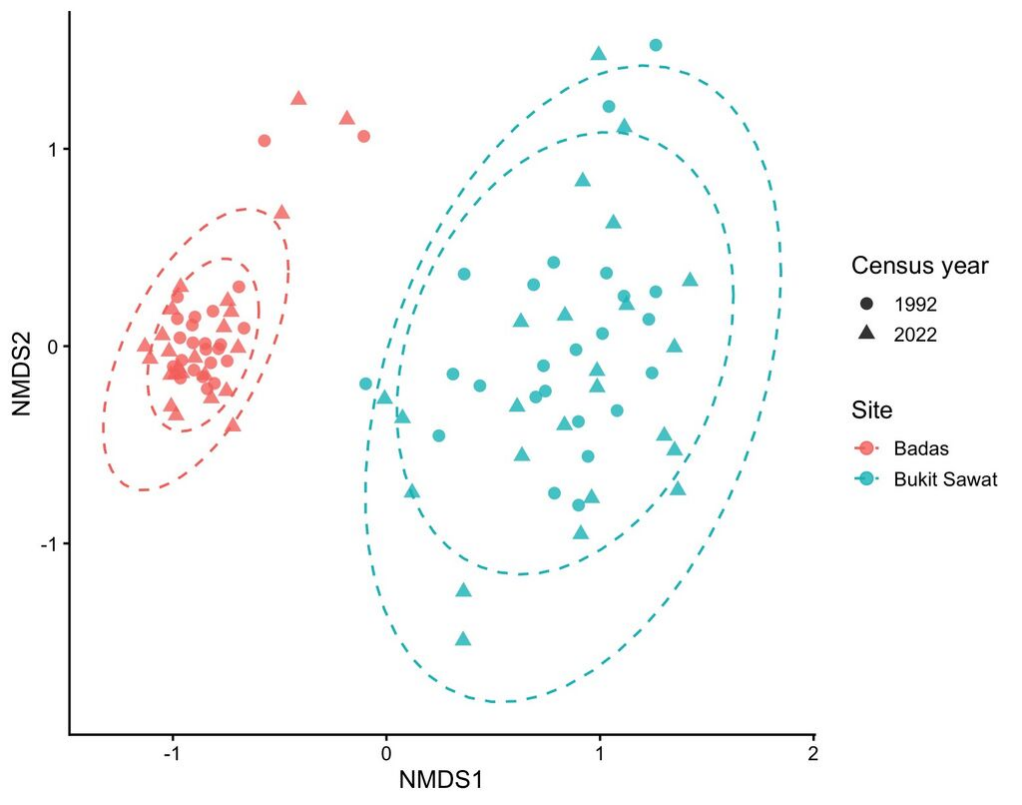
Non-metric multidimensional scaling (NMDS) ordination of tree community composition in two 0.96 ha permanent heath forest plots at Bukit Sawat and Badas, Brunei Darussalam, based on species abundance data from the 1992 and 2022 censuses. Each point represents an individual 20 × 20 m subplot. Symbols indicate census year (circles = 1992; triangles = 2022), while colours indicate study site (Badas and Bukit Sawat). Dashed ellipses represent 95% confidence intervals around group centroids. The ordination illustrates differences in tree community composition between sites and temporal shifts in community composition over the 30-year study period.

NMDS ordinations revealed differences in community composition between the two sites and between the two census periods at each site (Fig. 3). This is further supported by the PERMANOVA results which showed that tree communities differed significantly between the two census periods at Bukit Sawat (R² = 0.0629, p = 0.001) and Badas (R^2^ = 0.0614, p = 0.003; Table 3) (Table 3).

### Differences in growth, recruitment and mortality of trees between Bukit Sawat and Badas

The mean annual diameter growth rate (AGR), mean % mortality and mean % recruitment of censused trees were slightly higher in Badas (0.18 ± 0.011 cm year^-1^; 2.80 ± 0.15% year^-1^; 1.94 ± 0.17% year^-1^, respectively; Table 4) than Bukit Sawat (0.1 ± 0.015 cm year^-1^; 2.56 ± 0.23% year^-1^; 1.83 ± 0.25% year^-1^, respectively; Table 4). However, these differences between the two heath forests plots in mean AGR, mean % mortality and mean % recruitment were not significant (P > 0.05; Table 4, Suppl. material 4).

## Discussion

### Temporal changes in forest structure at the heath forests in Bukit Sawat and Badas

Over the 30-year period, both heath forest plots in Bukit Sawat and Badas recorded decreased mean stem abundances and decreased mean tree densities. This is consistent with higher mean % mortality of trees at both sites (Bukit Sawat = 2.52%; Badas = 2.77%) compared to mean % recruitment of new trees (Bukit Sawat = 1.86%; Badas = 1.98%), which likely contributed to decreased stem abundance. The decrease in stem abundance was higher in Badas (21.6% decrease) than in Bukit Sawat (18.3% decrease), consistent with the higher mean % mortality in Badas than Bukit Sawat. Despite both sites being located in protected areas, these significant decreases in stem abundance may be associated with disturbance events that occurred over the last 30 years. At Bukit Sawat, sub-plots located at the edge of the 0.96 ha plot had lower stem abundance than adjacent sub-plots in the forest interiors. Increased tree mortality and damage at forest edges are well known due to edge effects (Laurance and Curran 2008, Qie et al. 2017, Silvério et al. 2018). Forest edges in tropical forests have also been associated with increased tree fall resulting in elevated mortality of small-sized neighbouring trees (Laurance 2004, D'Angelo et al. 2004, Laurance and Curran 2008).

Additionally, a higher abundance of large trees with DBH ≥ 40 cm were recorded in the 2022 census than in the original 1992 census for both heath forest sites. This likely reflects forest growth processes over the 30-year period as the smaller trees from the 1992 census grow into larger trees, particularly in dominant large-sized tree families, such as the Dipterocarpaceae (Limin et al. 2021). Our results also showed that mean tree density was significantly lower, while both mean DBH and basal area were significantly higher at the most recent census than the original census. Comparable patterns have been observed in other long-term studies of tropical forests, showing declines in tree densities and increases in basal area over time (Lang and Knight 1983, Manokaran and Kochummen 1987, Korning and Balslev 1994, Newbery et al. 1999). Additionally, self-thinning processes during natural succession in forest ecosystems often result in decreased tree density, but increased basal area (Oliveira-Filho et al. 1997, Guariguata and Ostertag 2001, Long et al. 2025).

Forest structure can also be influenced by environmental variation and previous work conducted within the same permanent plots (Ikbal et al. 2024) demonstrated significantly higher canopy openness at Badas than at Bukit Sawat. These differences in canopy structure may contribute to local environmental heterogeneity and potentially influence some of the forest structure differences detected in the present study. However, canopy openness was not assessed during the 1992 census and, therefore, could not be directly evaluated here.

### Temporal changes in tree species richness, diversity and community composition at the heath forests in Bukit Sawat and Badas

Our findings show that, over the 30-year period, the heath forest tree communities at both sites have significantly changed, with the Bukit Sawat tree community appearing to have gained species, whereas the Badas tree community lost species. We recorded a 24% increase in total species richness in Bukit Sawat (i.e. 208 species in 2022 vs. 168 species in 1992) and a 4% decrease in total species richness in Badas (i.e. 108 species in 2022 vs. 113 species in 1992). Species turnover was evident at both sites, with 83 species newly recorded at Bukit Sawat and 36 species newly recorded in Badas in 2022, while several species recorded in 1992 were absent in 2022 (n = 53 species at Bukit Sawat, n = 46 species at Badas). Tree community composition differed between census periods at each site, as evidenced by directional shifts in the NMDS ordination space and significant PERMANOVA results for both sites. Additionally, our NMDS ordination also revealed a clear difference in tree community composition at Bukit Sawat and Badas. Differences in tree communities between Bukit Sawat and Badas for the 2022 census have previously been attributed to environmental variables, particularly topographic variables for Bukit Sawat and canopy openness for Badas (Ikbal et al. 2024).

Long-term studies of permanent plots in tropical forests have also shown similar patterns of increases or decreases in species richness and diversity and have been attributed to various factors such as environmental changes (Hubbell and Foster 1986, Tan et al. 2009) natural disturbance events (Peres 1999, Nakagawa et al. 2012), droughts (Dai 2013, Feng et al. 2013, Miyamoto et al. 2021) and anthropogenic disturbance, such as logging and land-use changes (Nakagawa et al. 2012, Cole et al. 2015, Rozak et al. 2018, Mahayani et al. 2022). Of these, we suggest that the effects of two major drought events (the 1997-1998 El Niño drought and the 2015-2016 El Niño drought), which occurred during the 30-year study period, may have had the greatest impact on tree dynamics, species richness and diversity and largely helped to shape the observed shifts in tree community compositions at our two study sites. Drought events are known to significantly impact forest structure, species composition and tree dynamics of Bornean tropical forests, including heath forests which are generally considered more vulnerable to prolonged drought and associated fire events (Nakagawa et al. 2000, Potts 2003, Newbery and Lingenfelder 2004, Itoh et al. 2012, Rifai et al. 2019, Miyamoto et al. 2021). Multiple fire events were reported in the surrounding areas of the Bukit Sawat and Badas plots during the 1997-1998 El Niño drought (Forestry Department, pers. comms.).

Additionally, anthropogenic disturbances could have influenced changes in tree communities at our plots. Notably, at Bukit Sawat, we recorded signs of increased disturbance within the plot's immediate vicinity, as indicated by the presence of invasive *Acacia mangium* and several species typical of secondary habitats, such as several *Macaranga* species, at the plot edges. Additionally, the Bukit Sawat plot is in close proximity to the Labi Road (approximately 100 m away), where *Acacia mangium* is commonly observed along the roadsides (Jaafar et al. 2022). *Acacia mangium* and other *Acacia* species have been recorded as invasive in Brunei's heath forests (Jambul et al. 2020, Tuah et al. 2020). Thus, the presence of *Acacia mangium* and proximity to roadside habitats suggest potential disturbance influences that may further contribute to changes in tree community composition over the 30-year period and will likely continue to exert further influence on the tree community at Bukit Sawat over time. At Badas, changes in the tree community composition may be related to increased disturbance events at the Badas peat dome (Addly et al. 2022) as the Badas plot is located on top of this peat dome and close to the Badas pipeline road (i.e less than 1 km in distance). Evidence of peat degradation resulting in peat subsidence and significant lowering of peat groundwater level had been reported from Badas, causing peat and small fires (Suhip et al. 2020, Addly et al. 2022). Peat subsidence and hydrological changes are known to significantly alter peat characteristics and environmental conditions (Hooijer et al. 2012) and these accumulated changes over a 30-year period at Badas could have potentially contributed to the observed changes in tree community composition.

### Variation in the growth, recruitment and mortality of trees

No significant between-site differences were recorded in mean annual growth rate (AGR), mean annual mortality rate and mean annual recruitment rate, indicating that both heath forest plots had similar growth, recruitment and mortality rates over the 30-year period. The mean annual mortality rates (Bukit Sawat = 2.56 ± 0.23%; Badas = 2.80 ± 0.15%) and mean annual recruitment rates (Bukit Sawat = 1.83 ± 0.25%; Badas = 1.94 ± 0.17%) were comparable with ranges reported for other tropical forests (Manokaran and Kochummen 1987, Korning and Balslev 1994, Newbery et al. 1999, Sawada et al. 2015, Ediriweera et al. 2020) Within Borneo, our mean annual mortality and mean recruitment rates were comparable to those recorded from the Andulau mixed dipterocarp forest site in Brunei (MDF; rates of 1.15% per year) (Limin et al. 2021), but lower than those from the Sebangau peat swamp forest in Central Kalimantan (rates of 0.02% - 0.05% per year) (Atikah 2014). Similarly, our mean annual mortality rate was comparable to the range of 1.8% to 5.8% recorded during the pre-drought periods in the tropical lowland heath forests in Nabawan, Sabah (Miyamoto et al. 2021).

### Limitations and recommendations

Although we postulate that several disturbance-related factors, including drought, fire, edge effects, *Acacia* invasion and peat degradation, may have contributed to the observed patterns of forest structure and changes in tree communities in our study, we acknowledge that these variables were not directly quantified and could not be statistically tested as causal effects. We therefore recommend that future research in these permanent heath forest plots at Bukit Sawat and Badas incorporate long-term climatic and hydrological monitoring and the quantification of disturbance causes, frequency and intensity, to help elucidate the mechanisms underlying temporal changes in heath forest structure and composition.

## Conclusions

This study provides evidence of temporal changes over a 30-year period in forest structure, species richness and diversity and tree community composition of two contrasting heath forest sites in Brunei Darussalam. Although both plots were established in intact forests within protected areas, past drought events and man-made disturbances likely influenced overall forest structure and tree community compositions. Given the threats to tropical forests posed by climate change, the present study is a crucial first step in improving understanding of the long-term dynamics of Bornean heath forests and their responses to a changing climate.

## Supplementary Material

CC68A3A2-56A0-5621-B019-84D9BA298F64 10.3897/BDJ.14.e194507.suppl1 Supplementary material 1 Forest structure comparison between the 1992 and 2022 censuses at Bukit Sawat and Badas heath forest plots: linear mixed effects model outputs Data type etc. Brief description ANOVA results from linear mixed‐effects models comparing forest structure between the 1992 and 2022 censuses at Bukit Sawat and Badas heath forest plots. File: oo_1672437.docx https://binary.pensoft.net/file/1672437 Irsalina S. Ikbal, Rahayu S. Sukri, Salwana Md Jaafar, Norhayati Ahmad

996303DF-999F-5402-84CB-82BEF0C00010 10.3897/BDJ.14.e194507.suppl2 Supplementary material 2 Tree species checklist, metadata and supporting documentation for the Bukit Sawat and Badas permanent heath forest plots (1992 and 2022 censuses) Data type Compressed archive (ZIP) Brief description Archive containing the tree species checklist and abundance records for the two 0.96 ha permanent heath forest plots at Bukit Sawat and Badas, Brunei Darussalam, based on the 1992 and 2022 censuses. The archive includes a dataset file, data dictionary and README document describing dataset contents, variable definitions, abbreviations, site codes, census years and data structure to facilitate interpretation and reuse of the dataset. Stem-level data are available upon request from the senior corresponding author (RSS). File: oo_1687256.zip https://binary.pensoft.net/file/1687256 Irsalina S. Ikbal, Rahayu S. Sukri, Salwana Md. Jaafar, Norhayati Ahmad

979C2849-35F1-53EE-AD0F-D29B6508DE6B 10.3897/BDJ.14.e194507.suppl3 Supplementary material 3 Species richness and diversity comparisons between the 1992 and 2022 censuses: linear mixed effects model outputs Data type etc. Brief description ANOVA results from linear mixed‐effects models testing differences in species richness and diversity indices between the 1992 and 2022 censuses at Bukit Sawat and Badas. File: oo_1672438.docx https://binary.pensoft.net/file/1672438 Irsalina S. Ikbal, Rahayu S. Sukri, Salwana Md. Jaafar, Norhayati Ahmad

693E5F66-39DC-5BC5-BC83-14B58159BEC4 10.3897/BDJ.14.e194507.suppl4 Supplementary material 4 Tree growth, mortality and recruitment rates in Bukit Sawat and Badas heath forest plots: linear mixed effects model outputs Data type etc. Brief description ANOVA results from linear mixed‐effects models testing between-site differences in mean diameter growth rate (AGR), mortality and recruitment at the Bukit Sawat and Badas heath forest plots. File: oo_1672439.docx https://binary.pensoft.net/file/1672439 Irsalina S. Ikbal, Rahayu S. Sukri, Salwana Md. Jaafar, Norhayati Ahmad

## Figures and Tables

**Table 1.** Differences in forest structure (mean stem abundance, mean tree density, mean diameter at breast height; DBH, basal area), total abundance, total basal area of trees in the two 0.96 ha plots at the heath forests at Bukit Sawat and Badas in 1992 and 2022. Mean values (± SE) were calculated over the total number of sub-plots per 0.96 ha plot at each site. *, ** and *** indicate a significant difference at p < 0.05, p < 0.01 and p < 0.001, respectively, as analysed using linear mixed effects (LME) models. Total abundance was calculated as the total number of censused stems within each 0.96 ha plot. Total basal area (m^2^) of a plot was calculated from the sum of basal areas of all censused trees within each 0.96 ha plot. Total tree density was calculated as the total number of censused stems divided by 0.96 ha plot.

	Bukit Sawat		Badas	
	1992 census	2022 census	1992 census	2022 census
Mean stem abundance	67.2 ± 2.8	54.9 ± 2.2**	55.9 ± 2.3	43.8 ± 1.8***
Mean tree density	1680.2 ± 69.2	1371.9 ± 55.7**	1396.9 ± 58.2	1095.8 ± 45.9***
Mean DBH (cm)	13.29 ± 0.33	14.17 ± 0.23*	13.5 ± 0.46	15.22 ± 0.50*
Mean basal area (cm^2^)	234.46 ± 16.4	284.31 ± 14.1*	278.85 ± 21.9	370.92 ± 29.3**
Total abundance	1613	1317	1341	1052
Total tree density	1680	1372	1397	1096
Total basal area (m^2^)	36.55	37.12	35.74	37.65

**Table 2.** Total species richness, mean species richness and mean diversity indices (Shannon’s Index, Inverse Simpson’s Index and Evenness) in the two 0.96 ha plots at the heath forests at Bukit Sawat and Badas in 1992 and 2022. Mean values (± SE) were calculated over the total number of sub-plots per 0.96 ha plot at each site. *, ** and *** indicate a significant difference at p < 0.05, p < 0.01 and p < 0.001, respectively, as analysed using linear mixed effects (LME) model. Total species richness was calculated from the sum of tree species recorded within each 0.96 ha plot.

	Bukit Sawat		Badas	
	1992 census	2022 census	1992 census	2022 census
Total species richness	168	208	113	108
Mean species richness	37.83 ± 1.41	32.04 ± 0.94**	21.08 ± 1.14	17.88 ± 1.14
Shannon’s Index	3.41 ± 0.04	3.25 ± 0.04*	2.55 ± 0.06	2.46 ± 0.07
Evenness	0.94 ± 0.01	0.94 ± 0.01	0.84 ± 0.009	0.86 ± 0.01
Inverse Simpson’s Index	24.61 ± 1.21	20.93 ± 1.00*	9.24 ± 0.82	9.03 ± 0.76

**Table 3.** Pairwise PERMANOVA result test results, based on abundance data, to show pairwise differences in tree community composition at each location over the 30-year period (1992 vs. 2022). Significant p-values are highlighted in bold.

Pairwise comparison	*F*	*R* ^2^	p
Bukit Sawat 1992 vs. Bukit Sawat 2022	3.09	0.0629	**0.001**
Badas 1992 vs. Badas 2022	3.01	0.0614	**0.003**

**Table 4.** Differences in mean annual diameter growth rate; AGR, mean mortality and mean recruitment of trees over the 30-year period (1992 vs. 2022) between the two 0.96 ha heath forest plots at Bukit Sawat and Badas. Mean values (± SE) were calculated over the total number of sub-plots per 0.96 ha plot at each site. *, ** and *** indicate a significant difference at p < 0.05, p < 0.01 and p < 0.001, respectively, as analysed using linear mixed effects (LME) models.

	Bukit Sawat	Badas
Mean AGR (cm/yr)	0.17 ± 0.015	0.18 ± 0.027
Mean mortality (%/yr)	2.52 ± 0.24	2.77 ± 0.14
Mean recruitment (%/yr)	1.86 ± 0.32	1.98 ± 0.23
