# Supplementary material for: Long-term dynamics of tropical heath forests in Brunei Darussalam: forest structure, tree demography and community reassembly over 30 years
Source: Biodivers Data J. 2026 Jun 30;14:e194507. doi: 10.3897/BDJ.14.e194507 (PMC13342929; doi:10.3897/BDJ.14.e194507)
Supplement: Supplementary material 1 — Forest structure comparison between the 1992 and 2022 censuses at Bukit Sawat and Badas heath forest plots: linear mixed effects model outputs [file bdj-14-e194507-s001.docx]

Table S1. Results of ANOVA from linear mixed effects model analysis showing differences in forest structure (stem abundance, tree density, diameter at breast height (DBH) and basal area) between the two censuses (1992 vs. 2022) at Bukit Sawat and Badas heath forest plots, respectively. Significant p-values at α = 0.05 are highlighted in bold.

|  | Mean abundance | | | Mean tree density | | | Mean DBH | | | Mean basal area | | |
| --- | --- | --- | --- | --- | --- | --- | --- | --- | --- | --- | --- | --- |
|  | dF | F | p-value | dF | F | p-value | dF | F | p-value | dF | F | p-value |
| Bukit Sawat | 1 | 12.06 | **0.002** | 1 | 12.06 | **0.002** | 1 | 4.79 | **0.039** | 1 | 5.32 | **0.03** |
| Badas | 1 | 16.52 | **< 0.001** | 1 | 16.52 | **< 0.001** | 1 | 7.13 | **0.014** | 1 | 8.58 | **0.008** |

Abbreviations: dF = degrees of freedom; DBH = diameter at breast height; F = F-statistic.
