# Supplementary material for: Long-term dynamics of tropical heath forests in Brunei Darussalam: forest structure, tree demography and community reassembly over 30 years
Source: Biodivers Data J. 2026 Jun 30;14:e194507. doi: 10.3897/BDJ.14.e194507 (PMC13342929; doi:10.3897/BDJ.14.e194507)
Supplement: Supplementary material 3 — Species richness and diversity comparisons between the 1992 and 2022 censuses: linear mixed effects model outputs [file bdj-14-e194507-s003.docx]

Table S3. Results of ANOVA from linear mixed effects model analysis of species richness and diversity indices (Shannon’s index, Evenness and Inverse Simpson’s index) showing the effects of two different census periods at the Bukit Sawat and Badas heath forest plots. Significant p-values at α = 0.05 are highlighted in bold.

|  | Shannon’s index | | | Evenness | | | Inverse Simpson’s index | | | Species richness | | |
| --- | --- | --- | --- | --- | --- | --- | --- | --- | --- | --- | --- | --- |
|  | dF | F | p-value | dF | F | p-value | dF | F | p-value | dF | F | p-value |
| Bukit Sawat | 1 | 7.70 | **0.011** | 1 | 0.01 | 0.913 | 1 | 5.80 | **0.024** | 1 | 12.77 | **0.002** |
| Badas | 1 | 0.95 | 0.34 | 1 | 3.27 | 0.08 | 1 | 0.04 | 0.84 | 1 | 3.94 | 0.059 |

Abbreviations: dF = degrees of freedom; F = F-statistic. Shannon's index, Evenness and Inverse Simpson's index were calculated at subplot level
