# Supplementary material for: Long-term dynamics of tropical heath forests in Brunei Darussalam: forest structure, tree demography and community reassembly over 30 years
Source: Biodivers Data J. 2026 Jun 30;14:e194507. doi: 10.3897/BDJ.14.e194507 (PMC13342929; doi:10.3897/BDJ.14.e194507)
Supplement: Supplementary material 4 — Tree growth, mortality and recruitment rates in Bukit Sawat and Badas heath forest plots: linear mixed effects model outputs [file bdj-14-e194507-s004.docx]

Table S4. Results of ANOVA from linear mixed effects model analysis for between-site differences in mean absolute diameter growth rate (AGR), mean % mortality and mean % recruitment at the Bukit Sawat and Badas heath forest plots, at α = 0.05 level.

| Effects | Mean AGR | | | Mean % mortality | | | Mean % recruitment | | |
| --- | --- | --- | --- | --- | --- | --- | --- | --- | --- |
|  | dF | F | p-value | dF | F | p-value | dF | F | p-value |
| Site | 1 | 0.092 | 0.813 | 1 | 0.801 | 0.380 | 1 | 0.090 | 0.767 |

Abbreviations: AGR = absolute diameter growth rate (cm year⁻¹); dF = degrees of freedom; F = F-statistic.
